# Supplementary material for: NRF2 activators inhibit influenza A virus replication by interfering with nucleo-cytoplasmic export of viral RNPs in an NRF2-independent manner
Source: PLoS Pathog. 2023 Jul 17;19(7):e1011506. doi: 10.1371/journal.ppat.1011506 (PMC10374058; doi:10.1371/journal.ppat.1011506)
Supplement: S1 Methods — (DOCX) [file ppat.1011506.s013.docx]

**S1 Methods (contains Sa-e Figs)**

**NRF2 activators inhibit influenza A virus replication by interfering with**

**nucleo-cytoplasmic export of viral RNPs in an NRF2-independent manner**

Fakhar H. Waqas1,2, Mahmoud Shehata3,4, ¶, Walid A.M. Elgaher5, ¶, Antoine Lacour5,10, ¶, Naziia Kurmasheva6, Fabio Begnini7, Anders E. Kiib7, Julia Dahlmann8,9, Chutao Chen1,2, Andreas Pavlou10, Thomas B. Poulsen7, Sylvia Merkert8,9, Ulrich Martin8,9, Ruth Olmer8,9,, David Olagnier6, Anna K.H. Hirsch5,11, Stephan Pleschka3,12, Frank Pessler1,2,13,*

**Table of contents**

**Item** **Page**

S1 Methods 2

Sa_Fig 3

Sb_Fig 4

Sc_Fig 4

Sd_Fig 5

Se_Fig 5

References 6

## **S1 Methods**

## **Organic synthesis – general methods**

All reactions were conducted in flame-dried glassware under an atmosphere of dry argon unless otherwise stated. CH2Cl2 was dried over activated 4Å molecular sieves and reagents were used as received from commercial suppliers (Sigma Aldrich, TCI and Fluorochem). Concentration in vacuo was performed using a rotary evaporator with the water bath temperature at 40 °C, followed by further concentration using a high vacuum pump. TLC analysis was carried out on silica coated aluminum foil plates (Merck Kieselgel 60 F254). The TLC plates were visualized by UV irradiation and/or by staining with KMnO4 stain. Purification was performed by automated flash column chromatography (AFCC) using an Interchim PuriFlash 420 instrument with 30 µm prepacked columns. Infrared spectra (IR) were acquired on a PerkinElmer Spectrum TwoTM UATR. Mass spectra (HRMS) were recorded on a Bruker Daltonics MicrOTOF time-of-flight spectrometer. Nuclear magnetic resonance (NMR) spectra were recorded on a Bruker BioSpin GmbH 400 MHz spectrometer, running at 400 and 101 MHz for 1H and 13C, respectively. The residual peak of the respective solvent was used as the internal standard: DMSO-*d*6 (CD2HSOCD3 *δ*H 2.50 ppm, CD3SOCD3 *δ*C 39.5 ppm).

Synthesis of *2-Methylene-4-(oct-7-yn-1-yloxy)-4-oxobutanoic acid* (**4-OI-alk**)

Itaconic anhydride (100 mg, 0.89 mmol, 1.0 eq.) was dissolved in anhydrous CH2Cl2 (0.5 mL). 7-Octyn-1-ol (338 mg, 2.67 mmol, 3.0 eq.) and conc. H2SO4(5 drops) were added, and the reaction mixture was stirred for 16 hours at r.t. Et2O (25 mL) was then added to the reaction mixture and the organic phase was washed with an aqueous solution of K2CO3 (10 w%, 2 × 10 mL). The aqueous phase was then extracted with Et2O (2 × 20 mL) to remove the non-ionizable impurities. Conc. HCl was then added to the aqueous phase until pH = 1 and
the aqueous phase was then extracted with CH2Cl2 (2 × 25 mL). The combined organic phases were dried over Na2SO4, filtered, and then concentrated under reduced pressure. The crude product was purified by flash chromatography on a silica gel column using 10-100% EtOAc in heptane as eluent to give **4-OI-alk** (70 mg, 0.30 mmol, 33%) as a white solid.

R*f =* 0.50 (Pentane/EtOAc 2:1; UV (254 nm) and KMnO4

HRMS (ESI) m/z calcd for C13H17O4 [M-H]- 237.1132, found 237.1131

IR νmax (cm-1) 3294, 2939, 1735, 1698, 1634, 1432, 1160, 960, 635 ννν

1H NMR (400 MHz, DMSO-*d*6) *δ* 12.62 (s, 1H), 6.15 (d, *J* = 1.6 Hz, 1H), 5.76 (d, *J* = 1.5 Hz, 1H), 4.00 (t, *J* = 6.6 Hz, 2H), 3.30 (s, 2H), 2.75 (t, *J* = 2.7 Hz, 1H), 2.15 (td, *J* = 6.9, 2.7 Hz, 2H), 1.58 – 1.49 (m, 2H), 1.46 – 1.39 (m, 2H), 1.43 – 1.22 (m, 4H).

13C NMR (101 MHz, DMSO-*d*6) *δ* 171.0, 167.8, 135.3, 128.4, 85.0, 71.7, 64.5, 37.7, 28.4, 28.3, 28.2, 25.2, 18.1.

*The NMR spectra (Sa-e Figs) are in agreement with the values reported in ref. (1).*

**Sa Fig:** 1H NMR spectrum (400 MHz, DMSO-*d*6) of **4-OI-alk**

**Sb Fig:** 13C NMR spectrum (101 MHz, DMSO-*d*6) of **4-OI-alk**

**Sc Fig**: COSY spectrum (400 MHz, DMSO-*d*6) of **4-OI-alk**

**Sd Fig**: HSQC NMR spectrum (400/101 MHz, DMSO-*d*6) of **4-OI-alk**

**Se Fig**: HMBC NMR spectrum (400/101 MHz, DMSO-*d*6) of **4-OI-alk**

**Reference**

1. Sun Q, Carrasco YP, Hu Y, Guo X, Mirzaei H, Macmillan J, et al. Nuclear export inhibition through covalent conjugation and hydrolysis of Leptomycin B by CRM1. Proc Natl Acad Sci U S A. 2013;110(4):1303-8.
