# Supplementary material for: NRF2 activators inhibit influenza A virus replication by interfering with nucleo-cytoplasmic export of viral RNPs in an NRF2-independent manner
Source: PLoS Pathog. 2023 Jul 17;19(7):e1011506. doi: 10.1371/journal.ppat.1011506 (PMC10374058; doi:10.1371/journal.ppat.1011506)
Supplement: Attachment — Submitted filename: Response file Waqas et al R1.pdf [file ppat.1011506.s015.pdf]

**Response file Waqas et al.**	
Major issues, key experiments	
**Reviewer 1**	
The authors examined three NRF2 activators, and found that the anti-IAV activity was correlated with their binding energies to XPO1. Are there any other NRF2 activators, if so, whether their anti-IAV activity all depends on their ability to bind XPO1 rather than acting as NRF2 activators. Is the antiviral effect of these NRF2 activators specific to IAV? How about their effect on other viruses that do not have a phase of replication in the nucleus?	We address this query in new text in Discussion (lines 340-347)
2. The authors demonstrated the inhibitory effect of these NRF2 activators on the nuclear export of vRNP complex of IAV. How about their effect on the nuclear export of host cellular proteins. To exclude potential toxic effect, the safe concentration range of the compounds in cells should be provided.	The safe concentrations of BARD, SFN and SEL were taken from the literature. We added this information to Methods (lines 428-430).<br><br>We assessed the impact of the compounds on p53 localization in IAV infected A549 cells. The results show that 4OI favors nuclear expression of p53 nearly as efficiently as SEL, whereas BARD and SFN have only weak effects.<br>Lines 320-329<br>Lines 393-397<br><br>Figure S10<br>New Table S1 (summarizing viruses that require XPO1 for optimal infectivity)
3. To further confirm the conclusion that the four compounds inhibit nuclear export of vRNP complex, the authors had better exclude the possibility that these compounds may affect the vRNP complex activity of IAV by using a minigenome assay.	Detection of NP via immunofluorescence can not only be used to determine intracellular RNP localization, but also demonstrates that viral genome transcription and gene expression is not affected: otherwise NP would not be detectable. NP expression also depends on prior expression and functionality of the RdRp subunits. Therefore, in our opinion a quantitative analysis of RdRp transcription and replication activity via primer extension does not add principal information.
**Reviewer #2:**	
1) Experiments and Figure 3J:<br>- As NRF2 activators likely display their effects by binding and inactivating KEAP1, the expression of the mRNA of the latter	We checked KEAP1 expression using existing cDNA from the experiment and added the result to Figure 3.<br><br>Lines 190-194

should also be checked and provided in the heat map for the different conditions.	
2) Experiments and Figure 4:<br>- The authors show that the NRF2 KO cells support significantly higher viral titers, which are still more or less reduced in the presence of the four compounds. They should again analyse the mRNA amounts of KEAP1 and if they remain unchanged, the authors should knock-down KEAP1 with specific siRNAs and then evaluate the virus titers. According to the results provided in Figure 5 and Figure 6, the authors claim that unlike SEL, BARD is a better ligand for KEAP1 than for XPO1 and if so, in the absence of KEAP1, BARD might become more available for binding and inhibiting XPO1 and would thus reduce more efficiently the viral titers.	We checked KEAP1 expression using existing cDNA from the experiment and added the result to Figure 4.<br>Lines 217-220<br><br>Since KEAP1 was expressed, we then knocked it down and checked viral titers. One interesting observation was that in untreated cells, the KEAP1 knock-down reduced viral titers, which agreed nicely with an innate anti-influenza function of KEAP1/NRF2 signaling. Interestingly, NRF2 appears to affect IAV replication in these cells at a post-transcriptional step. The results are shown in Figure 4, S5.<br><br>Antiviral effects of the compound were independent of KEAP1 expression status. Specifically, the KEAP1 knock-down did not enhance the antiviral activity of BARD (Fig. 4H).<br><br>Lines 230-239
- Analysing the viral titers in conditions of NRF KO and XPO1 KD will provide additional data on the mechanism of action of the analysed compounds.	We knocked down XPO1 in the NRF2 KO ECs. The results are somewhat limited by the fact that the XPO1 knock down was only about 75% (mRNA) in these cells. However, the results do suggest that the proviral effect of XPO1 is stronger than the antiviral effect of NRF2. They also show that BARD and SFN respond differently to the double KO/KD than 4OI and SEL.<br><br>Lines 239-251<br>Figure S6
3) Experiments and Figure 5A & B:<br>- If 4OI binds, as does SEL, the same active sites on XPO1 and KEAP1, competition experiments between these two compounds should provide additional proofs.	We performed competition experiments using unlabeled SEL (a click-chemistry probe of SEL was not available). The results show nicely that SEL competes with 4OI for binding to XPO1 and also, but less efficiently, for binding to KEAP1. This agrees with the results of our ligand-target modeling studies.<br><br>Lines 259-261<br>Figures S7, S8

**Part III – Minor Issues: Editorial and Data Presentation Modifications**	
Reviewer #1:	
1. The shuttling of vRNP complex between cytoplasm and nucleus is critical for the replication of IAV. To offer informative background information, the authors could present the current research advances in the nuclear import and export of vRNP complex of IAV, especially the host factors involved except for XPO1.	We updated the text.<br><br>Lines 125-128
2. Page 5, Lines 143-145: Knock-down of XPO1 did not affect levels of IAV hemagglutinin (HA) mRNA or NP in the absence or presence of the four compounds (Fig. 3D, E). However, in Figure 3E, the intensity of ACTB in lanes 8-10 is lower than those in other lanes. Meanwhile, the expression of NP in SEL-treated XPO1 KD cells is also obviously lower than others. Are the provided images representative? The ratio of NP/ACTB had better to be presented by using the ImageJ software.	We performed a fresh blot using lysates from another replicate of the same experiment. The results are more consistent now.<br><br>Figure 3
3. All compounds reduced intracellular IFN responses. The lower mRNA level of IFIT1 and CXCL10 in the drug treatment group can be associated with the inhibition of IFN pathway by drug itself or the lower virus titer caused by the compound treatment. The ISRE reporter assay had better to be carried out to differentiate these possibilities.	It is correct that we cannot differentiate in this case, but at least for 4OI it is well known that it reduces IFN levels in non-infected cells (e.g. Waqas et al Clin Transl Med 2022). But we don't think the ISRE assay would help to differentiate between infection-dependent and -independent effects of the compounds, as the reporter would respond equally to signal changes driven by antiviral or anti-IFN effects of the compounds.<br>There is no good correlation between anti-IFN and anti-IAV effects of the compounds, but our data do not allow us to distinguish between infection-dependent and –independent effects of the compounds. The results probably reflect a mix of both. We added a sentence to Results explaining this.<br><br>Lines 253-254
4. Line 360: the authors carried out the immunofluorescence assay at 2, 4, 6 and 8 h p.i. However, the images of NP staining at 2 h p.i. were not shown in Fig. 2C.	The 2 h time point was actually not measured. This was a typo in Methods, which we have corrected.

5. How was the virus titer determined in Fig. 3F and 4B? What does FFU mean?	We clarified this in the respective figure legends.
**Reviewer #2**	
- The authors should explain the huge differences in the concentrations of the four compounds used, varying from 0.1 to 100 µM and which, I believe, are related to their cellular toxicity.	We clarified this in Methods<br><br>Lines 428-430
- In 2C the authors should indicate and discuss the percentage of infected (NP-expressing) cells for each condition, with or without treatment.	We measured the percentage of NP-expressing cells (using the original images) and added the results to Figure 2. SEL treatment led to significantly higher fraction of NP+ cells especially at 6 and 8 h p.i. (compared to the other groups). Possible causes are discussed in the text.<br><br>Lines 152-158<br>Figure S1
- IF staining of the different conditions is also needed at 0 h p.i, as control.	We added the 0 h. p.i. images to Figure 2.
**Part III – Minor Issues: Editorial and Data Presentation Modifications**	
**Reviewer #2**	
The authors should carefully check the references: references 1 and 14 are identical, the reference Qin et al. in the Supplemental Data is already cited in the main list as n°18.	We corrected the ref. 1 vs. 14 duplication. We changed the ref. Qin et al. in Supplement to Ref. 18 (main text)
