# Supplementary material for: NRF2 activators inhibit influenza A virus replication by interfering with nucleo-cytoplasmic export of viral RNPs in an NRF2-independent manner
Source: PLoS Pathog. 2023 Jul 17;19(7):e1011506. doi: 10.1371/journal.ppat.1011506 (PMC10374058; doi:10.1371/journal.ppat.1011506)
Supplement: Attachment — Submitted filename: PLOS_Pathogens_MD_comments.docx [file ppat.1011506.s014.docx]

Comments for the Authors

In this manuscript, Waqas *et al.* seek to shed light on the mechanisms regulating the antiviral effects of three NRF2 activators on IAV. They show that 4OI, SFN and BARD, as well as the potent CRM1 inhibitor SEL, inhibit more or less efficiently the nuclear export of IAV vRNPs. Surprisingly, they show that the compounds target, interact and probably inactivate CRM1, and that this inhibition is NFR2 independent. More interestingly, they provide ligand-target modeling data suggesting that the three NRF2 activators interact with different energies with the XPO1 NES-binding hydrophobic pocket. Moreover, the XPO1 inhibitor SEL can potentially interact in the same way with the active site of KEAP1, which is the main target of 4OI, SFN and BARD.

I have the following specific concerns:

1) Experiments and **Figure 2**:

- The authors should explain the huge differences in the concentrations of the four compounds used, varying from 0.1 to 100 μM and which, I believe, are related to their cellular toxicity.

- In **2C** the authors should indicate and discuss the percentage of infected (NP-expressing) cells for each condition, with or without treatment.

- IF staining of the different conditions is also needed at 0 hpi, as control.

2) Experiments and **Figure 3J**:

- As NRF2 activators likely display their effects by binding and inactivating KEAP1, the expression of the mRNA of the latter should also be checked and provided in the heat map for the different conditions.

3) Experiments and **Figure 4**:

- The authors show that the NRF2 KO cells support significantly higher viral titers, which are still more or less reduced in the presence of the four compounds. They should again analyse the mRNA amounts of KEAP1 and if they remain unchanged, the authors should knock-down KEAP1 with specific siRNAs and then evaluate the virus titers. According to the results provided in Figure 5 and Figure 6, the authors claim that unlike SEL, BARD is a better ligand for KEAP1 than for XPO1 and if so, in the absence of KEAP1, BARD might become more available for binding and inhibiting XPO1 and would thus reduce more efficiently the viral titers.

- Analysing the viral titers in conditions of NRF KO and XPO1 KD will provide additional data on the mechanism of action of the analysed compounds.

4) Experiments and **Figure 5A & B**:

- If 4OI binds, as does SEL, the same active sites on XPO1 and KEAP1, competition experiments between these two compounds should provide additional proofs.

Minor issues:

2) The authors should carefully check the references: references 1 and 14 are identical, the reference Qin *et al*. in the Supplemental Data is already cited in the main list as n°18…
